# A path toward disability-inclusive health in Zimbabwe Part 1: A qualitative study on access to healthcare

**DOI:** 10.4102/ajod.v11i0.990

**Published:** 2022-05-30

**Authors:** Tracey Smythe, Thubelihle Mabhena, Shepherd Murahwi, Tapiwanashe Kujinga, Hannah Kuper, Simbarashe Rusakaniko

**Affiliations:** 1 Department of Clinical Research, Faculty of Infectious and Tropical Diseases, London School of Hygiene & Tropical Medicine, London, United Kingdom; 2 Pan African Treatment Access Movement, Harare, Zimbabw; 3 Leonard Cheshire Disability Zimbabwe, Harare, Zimbabwe; 4 Department of Community Medicine, University of Zimbabwe, Harare, Zimbabwe

**Keywords:** disability, Zimbabwe, qualitative, equity, Missing Billion, inclusion, health system, health access

## Abstract

**Background:**

On average, people with disabilities have greater healthcare needs, yet face a range of barriers in accessing care.

**Objectives:**

Our objectives were to explore the experiences of people with disabilities in accessing care and identify opportunities for the health system to be designed for inclusion in Zimbabwe.

**Methods:**

In-depth qualitative interviews were conducted between May and June 2021 with 24 people with disabilities (identified through purposive sampling) and with 10 key informants from local and national health authorities (identified through expert recommendations). Interviews explored the experience of accessing healthcare prior to the coronavirus disease 2019 (COVID-19) pandemic. Interviews were transcribed, coded and thematically analysed. We used the disability-inclusive health ‘Missing Billion’ framework to map and inform barriers to inclusive healthcare and disparities in outcomes faced by people with disabilities.

**Results:**

People with disabilities experienced difficulties accessing health services in Zimbabwe prior to COVID-19. These experiences were shaped by health literacy, self-stigma and affordability of services, which limited demand. Supply of health services was constrained by the perceived poor capacity of health workers to treat people with disabilities and discrimination. Inclusion was facilitated by clinic staff support of people with disabilities’ access to medication through referral to mission hospitals and private clinics, and the lobbying of organisations of people with disabilities.

**Conclusion:**

Strategies to promote disability inclusion in healthcare include meaningfully engaging people with disabilities, investing in organisations of people with disabilities, protecting funding for disability inclusion, collecting and analysing disability-disaggregated data and strengthening a twin-track approach to health service provision.

## Background

There are approximately one billion people with disabilities globally. People with disabilities are a highly diverse group, including people with a range of impairment types, ages, genders and environments (WHO2011). However, across the spectrum, people with disabilities on average have additional general health needs and also often specific health needs relating to the person’s impairment (Kuper & Heydt 2019). Consequently, a recent systematic review highlighted that healthcare needs are greater for people with disabilities (Bright & Kuper 2018). Nevertheless, they also face barriers in accessing care, including financial, accessibility and skills and knowledge of healthcare workers (Bright, Wallace & Kuper 2018; Kuper, Smythe & Duttine 2018). People with disabilities therefore have higher mortality rates, worse coverage of services, incur higher healthcare costs and experience worse quality of care (Kuper & Heydt 2019; WHO 2011). This exclusion from healthcare access is a violation of their fundamental rights, as laid out in the UN Convention on the Rights of Persons with Disabilities (UN 2007), and will also make it difficult to reach Sustainable Development Goal 3 (UN 2015) to ‘ensure healthy lives and promote wellbeing for all at all ages’ and other health targets.

**FIGURE 1:**
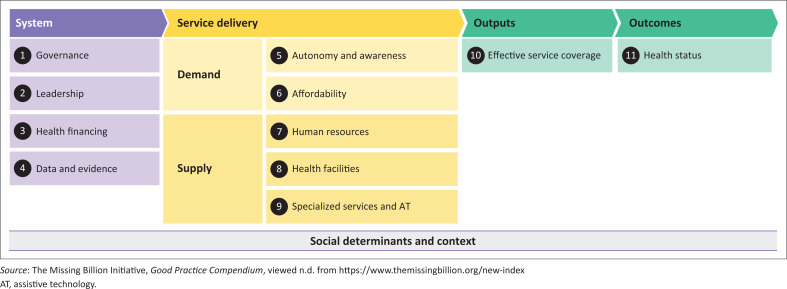
Preliminary framework of inclusive health systems.

Barriers in access to healthcare are likely to be greater for people with disabilities in low and middle-income countries (LMICs) (Banks et al. 2015; Prynn et al. 2021; Werfalli et al. 2018). In Zimbabwe, for example, the health delivery systems are experiencing important challenges following decades of under-investment (Kidia 2018), and the health systems are therefore in urgent need of strengthening. Here, people with disabilities should be a priority as they face stark health inequities, consistently have poorer health and difficulties receiving quality care (Eide et al. 2003b) because of the barriers that they face in seeking care (Muderedzi et al. 2017; Pata 2017; Rugoho & Maphosa 2017; Rukuni et al. 2018). The ‘Missing Billion’ Report (Hogan 2020; Litullo 2019) (Figure 1) provides a framework for how to identify components of the health system that require strengthening in order to provide disability-inclusive healthcare services. The framework proposes core service delivery components needed for inclusive health, from the perspective of people with disabilities – ‘demand’ (awareness and autonomy, affordability) and service providers – ‘supply’ (e.g. adequately skilled human resources, accessible health facilities, availability of specialised services and assistive technology). These service delivery components depend on the presence of supportive systems-level factors, including good governance and leadership on disability-inclusive health, adequate health financing and the availability of data and evidence. Improving these healthcare system components should result in better effective service coverage for people with disabilities and ultimately better health status. However, evidence is lacking for Zimbabwe on what the perceived challenges are to inclusive health, and key actions to improve accessibility.

Our qualitative study aimed to use participatory approaches to understand the experiences and perceptions of people with disabilities in accessing healthcare in Zimbabwe prior to the coronavirus disease 2019 (COVID-19) pandemic and identify opportunities for the health system to be designed for inclusion in Zimbabwe.

## Methods

### Study design

A qualitative study was undertaken in Zimbabwe (31 May–12 June 2021) in the capital city Harare and rural and urban areas of Gutu to collect data to build the evidence base on disability-inclusive healthcare in contrasting settings. The data collection focused on lived experiences and perceived challenges in accessing healthcare pre-pandemic and how these could be overcome.

### Participants and setting

Participants included 24 people with disabilities in Gutu and 10 key informants from local and national health authorities in Gutu and Harare. People with disabilities were recruited through non-governmental organisations (NGOs) and organisations of people with disabilities (OPDs) through purposive sampling. The NGOs and OPDs recommended information-rich cases and provided the researchers with a contact list. People with disabilities were subsequently selected to ensure representation by impairment type or condition (e.g. physical, sensory, intellectual), age (children, working-age, older adults), gender and level of support needed for daily life (e.g. none or minimal, ongoing healthcare or social service needs, requiring carer support for activities of daily living). Key informants were selected by expert recommendation based on their pivotal role and experience in disability programming. All participants were approached through telephone calls. Only two individuals who initially agreed to be interviewed when contacted through telephone calls were subsequently unable to take part in the interviews because of health-related concerns. Demographics of the 24 participants with disabilities are outlined in Table 1.

**TABLE 1:** Demographics of participants with disabilities by impairment category†, age and sex.

Category	Frequency (*n*)
**Impairment/condition** †	
Epilepsy	4
Intellectual/behavioural	4
Physical impairment	8
Speech and/or hearing impairment	5
Visual impairment	5
**Age (years)**	
< 10	2
10–20	3
21–30	5
31–40	3
41–50	2
50+	9
**Sex**	
Female	13
Male	11

† , 2 participants had more than one impairment.

In-person interviews were conducted at the homes of people with disabilities and at the place of work of key informants. The majority of participants were interviewed directly (face-to-face). However, carer or proxy interviews were used for children below the age of consent (aged 10 as per national guidelines) and for people with severe difficulties understanding or communicating even with available adaptations (e.g. people with hearing loss, illiterate and with no knowledge of sign language; people with severe intellectual or cognitive impairments). Children aged 10 years or older but below the age of consent participated in interviews with parental consent and individual assent.

Inclusion of people with disabilities was supported through the provision of psychological support services when needed, sign language interpretation, accessible interview sites and transport, use of available district psychological services and researchers skilled at communicating with people with cognitive impairments.

### Data collection

Interview guides with questions and prompts (Appendices 1 and 2) were developed and reviewed by 10 people with disabilities during a consultative workshop in April 2021. The topic guides were cognitively tested for understanding and administered in English or Shona by trained research assistants. The research assistants were three women with disabilities who had completed tertiary education. They underwent a one-day online training that included presentation of the study protocol and qualitative methods. Next, they attended a two-day in-person training on data collection, with ongoing mentoring and support provided by the study team (TM, SM, SR). No interviews were repeated and transcripts were not returned to participants for comment. Interviews took between 30 and 60 min and were audio recorded with written consent from the participants.

### Data management and analysis

All interviews were transcribed verbatim for analysis and translated into English where necessary. Data were managed using NVivo 12. Interview transcripts and detailed notes were analysed using a deductive thematic analysis (Guest, Macqueen & Namey, 2012). A coding framework was developed using the semi-structured interview guide as a starting point, which was adapted to include additional codes and themes emerging from the data. An allied health professional and epidemiologist from Zimbabwe with experience in both qualitative and quantitative research methods (TS) coded the interview transcripts to identify the key themes emerging from the data. These were discussed across the entire team, and analysis was evaluated by research team members (TM, SM, TK and SR) to ensure that interpretations were credible and valid. Regular discussions with the research team, including the research assistants (three women with disabilities), took place throughout the data analysis phase to ensure content validity and context.

We undertook a narrative synthesis of the findings and reported the results according to the consolidated criteria for reporting qualitative research (COREQ) (Tong, Sainsbury & Craig 2007), which is a 32-item checklist. We used the Missing Billion health system framework (Figure 1) (Hogan 2020; Litullo 2019) to map and inform barriers to healthcare and disparities in outcomes faced by people with disabilities. The predominant focus was on the service delivery components of the framework, including the demand and supply-side perspectives.

### Trustworthiness of the data and processes

Digital voice recordings were compared with transcriptions for accuracy. We used investigator triangulation, and TS, TM and SM made coding and analysis decisions based on a subset of the transcripts. The researchers involved in the interviews kept a record of daily activities concerning research and decisions influencing how the study was carried out. One researcher (TM) documented all steps and procedures used for a data audit trail to identify the potential for bias or distortion.

### Ethical considerations

Ethical approval for the study was granted from the Medical Research Council of Zimbabwe (MRCZ) (No MRCZ/A/2731) and the Institutional Review Board of London School of Hygiene & Tropical Medicine, UK (No 22138 – 2).

The three main ethical considerations included addressing participant expectations, data protection and sensitive information. We took care to describe the nature and detail of our study to avoid raising expectations of participants on the outcome of the interview process. We did not link any data to particular participants. An information sheet describing the study protocols, including data management processes, procedures for maintaining confidentiality and plans for data sharing, was given to participants during recruitment. This information was reiterated verbally as part of the informed consent process in the language of choice (English or Shona). Informed consent was sought before the start of all interviews and taken by the trained research assistants. The research assistants were trained on identifying the need for referral of services and were accompanied in the field by representatives of OPDs, should further assistance with referral be required. All interviewees were compensated for their time and transport was reimbursed.

## Results

We present the data under the five themes that comprise the Missing Billion framework (Kuper & Heydt 2019). These include demand and supply-side factors for service delivery: ‘demand’ (autonomy and awareness, affordability), ‘supply’ (human resources, health facilities, specialised services and assistive technology). Table 2 provides an overview of the themes and sub-themes identified.

**TABLE 2:** Overview of themes.

Themes	Sub-themes
**Demand**	
Autonomy and awareness	Health literacy related to general health and impairment specific needs Perception of benefits of attending health services Health beliefs and self-stigma
Affordability	Out-of-pocket payments Transport costs Equipment costs Opportunity costs in seeking care Access to medication
**Supply**	
Human resources	Healthcare provider knowledge and capacity Healthcare provider attitude Pathways to care OPD support
Health facilities	Distance to healthcare clinic Physical accessibility Resources available for reasonable accommodation
Specialised services and Assistive Technology	Ability to engage Satisfaction with care

OPD, organisations of people with disability.

### Demand – Autonomy and awareness

People with disabilities demonstrated a varied awareness of their health needs. One participant attempted multiple clinic visits for a diagnosis: ‘I also approached other doctors, I went to South Africa too’ (Person with disabilities 18). Another demonstrated long-term awareness and understanding of the implications for the economy and society:

‘If government excludes me from their health and education planning, then I will continue to depend on them and it will cost them.’ (Key Informant 02)

However, there was often a delay in seeking health services. Health literacy was an important factor that shaped the ability of people with disabilities to realise and acknowledge their health needs; when asked why there was a delay in seeking care, one participant reported, ‘I was just very ignorant’ (Person with disabilities 05).

People with disabilities conveyed some understanding of the benefits of attending health facilities for general and rehabilitative needs; however, this was limited by awareness of what services may be provided. In addition, where health services may have provided medical support and advice, the internalisation of the attitudes of health workers over medical issues that were perceived to be embarrassing (self-stigma) led to limited health-seeking behaviour. Explaining his reticence to seek care for incontinence, a participant stated:

‘I have never told anyone; I do not know how to talk about this.’ (Person with disabilities 18)

No participants, neither those living in rural nor urban areas, reported receiving ongoing education about prevention of non-communicable diseases or secondary complications to their functional impairment.

### Demand – Affordability

Access to healthcare was limited by lack of funds, in particular for equipment and transport. Caregivers expressed concerns for the health and development of children with disabilities, and one caregiver of a child with a disability stated:

‘I heard some people say he might need toys to play with, and at the same time exercise his body, as he might balance and stand using those toys. But l do not have money to buy the toys.’ (Caregiver of child with disabilities 16)

Funding for transportation was experienced as an additional pressure on resources, in terms of both finance and travel time to health facilities. This pressure was heightened when a service was not received and repeat visits were required:

‘The challenge was for me to go to the clinic and see for sure that the clinic didn’t have any of the medication. They usually don’t have it.’ (Person with disabilities 21)

‘I then went with him to the specialist in Harare Hospital. When I got there, they said the specialist was not there.’ (Person with disabilities 05)

Affordability of medication was potentially less of an issue. As examples, people with disabilities and living with HIV reported good access to antiretroviral medication (ARVs) prior to the COVID-19 pandemic, and people with albinism noted that they had some access to protective creams. Explaining this, participants stated:

‘I used to get tablets [ARVs] for six months from the clinic, I wouldn’t pay anything for it.’ (Person with disabilities 25)

‘Before Covid it was better…we were given a three-month supply of sunscreen and lip balm at each visit.’ (Person with disabilities 22)

People with disabilities were advised to buy medication from private pharmacies, but this was often unaffordable: ‘I have a persistent headache but I cannot get medication because it’s sold in US$ and I do not have that money’ (Person with disabilities 17). Nevertheless, they perceived clinics as supporting their access to medication through securing donors for medication, and giving advice about where it may be available, such as through attending mission hospitals or buying medication from private pharmacies. In addition, facilitating factors toward achieving equitable access to healthcare included accounts of service providers who described how clinics attempted to increase inclusion through some cost savings,

‘Our clinics in the district do not charge for their services.’ (Key informant 08)

### Supply – Human resources

People with disabilities reported that their needs were often not understood by health workers, and some believed that they are treated as ‘patients of low priority’. They perceived a lack of capacity amongst health workers to treat people with disabilities and negative attitudes about people with disabilities within the health system. For instance:

‘As a person with a disability, you cannot do some of the activities and health workers have no patience for that.’ (Person with disabilities 18)

They also experienced language and communication barriers:

‘If you want medical attention and have hearing loss or intellectual impairment or a speech impairment, the doctors will say that they want the patients to speak for themselves. The doctors won’t allow anyone to speak for the patient, yet the aide might be the only one who can really understand the patient.’ (Key Informant 3)

Poor referral systems and limited medical expertise compounded their experiences of discrimination, for example:

‘Sometimes they don’t even take time to address you because you have a disability.’ (Person with disabilities 18)

‘Some health workers fear us and prefer to call senior nurses to treat us. Even when we go for minor procedure like checking of one’s blood pressure, some health workers are afraid to touch us and sometimes it becomes very obvious that we are excluded.’ (Person with disabilities 22)

There was also variation in how people with disabilities experienced support within clinics. For example, some people with disabilities reported being recognised by clinic staff, and this was viewed in a positive light as ‘reasonable accommodation’ because people with visual impairment or older people may not have to wait in line:

‘I think it’s because of my condition, that is why I’m served quickly. I’m not sure about everyone else though.’ (Person with disabilities 17)

‘If you have a known disability, you are sometimes even served first.’ (Person with disabilities 12)

In addition, some people with disabilities reported being satisfied with services, in particular for general health needs where ‘they always gave me the attention that I needed’ (Person with disabilities 05).

However, this experience was not similar to others who perceived being treated differently because of their impairment:

‘If I am with this lady [indicates to the research assistant], she can be served and I will be told to wait and be served later.’ (Person with disabilities 22)

Support processes for reasonable accommodation were in place in some settings, but not necessarily actioned:

‘The general policy of the hospital is that they should be attending to the persons with disabilities as soon as they notice them in the queue, or help them get to wherever they want to be. That is the general policy of the hospital. But then I cannot really verify that everybody is following that.’ (Key informant 05)

Healthcare workers also recognised that they could not identify people with disabilities in need or interact with patients once they are home:

‘If they could reach us, we were able to assist especially, for example, in terms of ART provision.’ (Key informant 04)

Programmes therefore attempted to include people with disabilities in service planning:

‘We have tried to incorporate people with disabilities in all meetings at national level, provincial level, at district level and at ward level.’ (Key information 04)

People with disabilities viewed OPDs that lobbied for inclusion in planning services and social protection programmes as supportive of their needs. Nevertheless, there was little information shared by representatives of the government public health system with OPDs about how inclusion was planned for service provision, and in general only one OPD may be invited for consultation.

### Supply – Health facilities

Primary healthcare clinics were located near to all participants and people with disabilities predominantly used the government public health system to access healthcare and treatment. People with disabilities frequently experienced physical exclusion with inaccessible buildings, ‘there is a big challenge because there are no ramps, they have steps only … in clinics there are no disability friendly toilets’ (Person with disabilities 04), which required additional assistance of a caregiver to enable access. Infrastructure and physical barriers contributed to the exclusion of people with disabilities and limited their physical access to health services.

The accounts of key stakeholders highlight how health providers try to reconcile the limited resources with the priorities of patients. Key stakeholders unanimously described a desire to support people with disabilities to achieve good health outcomes. Their accounts are interwoven with descriptions of their perceived pressure on resources:

‘People with disabilities have different requirements, and if we check in terms of the budget indications, they need more support.’ (Key informant 04)

However, this has not translated to support of inclusive health facility infrastructure and physical environment in either rural or urban areas.

### Supply – Specialised services and assistive technology

Specialist services required additional travel to larger towns with provincial hospitals (secondary and tertiary institutions). People with disabilities regularly experienced organising transport and finances to attend appointments only to find that the specialist was not in attendance or available on that day:

‘We had gone to a provincial hospital and unfortunately the specialist was not there, and because [*name*] was not feeling well during that time, we were referred to a specialist psychiatric hospital. The person who was with me called an ambulance. We rushed to the specialist hospital and the doctor was not there again.’ (Caregiver of person with disabilities 01)

Poor team communication between specialists and rehabilitation providers and limited explanation of the purpose and beneficial use of assistive technology affected the mental health and wellbeing of patients, whose experiences were characterised by worry and concern for their own health.

### Outcomes and impact on functioning

People with disabilities did not have sufficient funds to continue to attempt to access medical care or treatment following unsuccessful attempts. This often resulted in worsening of their functional impairment and secondary complications. After experiencing worsening pain and repeated hospital visits, one person explained, ‘because of the situation that I was in, I lost hope.’ (Person with disabilities 18)

The experience of repeated unsuccessful attempts to access healthcare was viewed as discouraging and the experience was underpinned by a loss of hope and confidence in the health system. There was limited ability to be empowered and minimal opportunity to engage in one’s own health journey.

These experiences and views demonstrate that the health needs of people with disabilities in Zimbabwe have not been protected, and the emergence of a pandemic presents further challenges given the competing demands and opportunities for the development and delivery of inclusive and equitable healthcare.

## Discussion

People with disabilities experienced difficulties in accessing health services in rural and urban areas of Zimbabwe prior to COVID-19. These barriers were experienced from the demand side, meaning the perspective of the person with disabilities, and included poor health literacy, lack of finances and self-stigma, where people with disabilities did not seek care for medical issues that were perceived to be embarrassing. Service-side challenges were also noted, including perceived poor capacity of health workers to treat people with disabilities, discrimination and inadequate health facilities. Outcomes for people with disabilities included decreased functioning and increased pain, and this experience was characterised by a lack of hope. Inclusion was perceived to be facilitated by clinic staff who supported people with disabilities’ access to medication through referral to mission hospitals and private clinics, and OPDs who lobbied for inclusion in planning services and social protection programmes.

Our key findings are similar to those in other LMICs. For example, health expenditure is typically higher for people with disabilities, and commonly reported barriers included those related to geographic accessibility, financial accessibility and acceptability of health services (Bright & Kuper 2018; Hannass-Hancock et al. 2017). In a recent meta-synthesis (Hashemi et al. 2020), authors found that the choice to seek health services by people with disabilities in LMICs, as well as the quality of intervention provided by primary healthcare providers, were influenced by cultural beliefs or attitudinal barriers, informational barriers, and practical or logistical barriers. Likewise, whilst there is a general lack of availability of rehabilitation services in LMICs (Gimigliano & Negrini 2017), a systematic review found that access to rehabilitation services for people with disabilities in LMICs was highly variable and poorly measured within the included studies, and access to rehabilitation services was generally low (Bright et al. 2018).

A path toward disability-inclusive health requires more than consideration of the potential barriers and perceived challenges to inclusive health; key actions to improve accessibility are needed. Demand and supply factors for health service delivery, as conceptualised by the Missing Billion framework (Hogan 2020; Kuper & Heydt 2020; Litullo 2019), need to be identified and improved. In Zimbabwe, there is a clear need for improved education on healthcare needs for people with disabilities, support to afford healthcare such as through social protection and tackling issues around self-stigma. These strategies have been employed in similar low-resourced settings (Kuper et al. 2020) with the growing global focus on disability-inclusive development.

From the healthcare provider perspective, actions are needed to ensure that medical protocols do not discriminate on the basis of disability, that medical facilities are accessible, including signage and information, and to promote healthcare worker awareness and inclusive attitudes (Shakespeare, Iezzoni & Groce 2009). These service delivery components will be supported by making systems-levels changes, which were not the focus of the current study. For instance, government departments should meaningfully engage people with disabilities in both rural and urban areas, or their representative organisations, to ensure an inclusive perspective to provision of healthcare (UN 2007). Supporting OPDs to advocate for inclusive health will improve support for people with disabilities. Strengthening a twin-track approach where people with disabilities are considered both in mainstream policy and in disability-specific policy will require protecting funding for disability inclusion across all healthcare initiatives (Dean et al. 2018). Collecting and analysing disability-disaggregated data will inform financial measures and economic planning (Abualghaib et al. 2019).

Our study has strengths and limitations which should be taken into account when interpreting the results. We achieved both breadth and depth of functional impairment and age range in our sample of participants. Nevertheless, this is a qualitative study of a modest-sized group of people with disabilities living in rural and urban areas of Zimbabwe, so it cannot be representative in the same way as a large-scale quantitative survey. Researchers with disabilities were trained to undertake the qualitative data collection, which likely improved data quality through strengthening the rapport of the interviewer and participant. However, the interviewers may also have brought their own biases to the interview, based on their personal experiences. In addition, all the transcripts were coded by a single coder; nevertheless, several checks were in place to strengthen the integrity of data and interpretations. These included involving research assistants who collected the interviews in data analysis and interpretation and ongoing discussions amongst the whole team throughout data collection and analysis, particularly on our positionality and reflexivity. The Missing Billion framework provided a structure for consideration of challenges and solutions to inclusive health. We used this framework to consider demand and supply side service delivery factors in this study, but did not address systems-level factors such as governance and leadership.

## Conclusion

People with disabilities are a diverse group and experience inequality in accessing health services in Zimbabwe. Strategies are needed to better protect the health needs of people with disabilities in Zimbabwe, including engaging with people with disabilities, investing in OPDs, protecting funding for disability inclusion, collecting and analysing disability-disaggregated data and strengthening a twin-track approach to health service provision. These actions have costs, but there is also a cost when people with disabilities are left behind (Banks & Polack 2014). Without concerted effort to significantly improve access to healthcare for people with disabilities, the goal of universal health coverage will not be achieved (Kuper & Hanefeld 2018). This exclusion is also a violation of their fundamental rights, as laid out in the UN Convention on the Rights of Persons with Disabilities (UN 2007). Finally, good health is fundamental to living a good life, including taking part in education and employment. The failure to include people with disabilities in healthcare means that we will fail to maximise their capabilities and contributions to society, and this will be to the detriment of all.
